# Replacement of Oxygen by Sulfur in Small Organic Molecules. 3. Theoretical Studies on the Tautomeric Equilibria of the 2OH and 4OH-Substituted Oxazole and Thiazole and the 3OH and 4OH-Substituted Isoxazole and Isothiazole in the Isolated State and in Solution

**DOI:** 10.3390/ijms17071094

**Published:** 2016-07-09

**Authors:** Peter I. Nagy

**Affiliations:** Center for Drug Design and Development, The University of Toledo, Toledo, OH 43606, USA; pnagy@utnet.utoledo.edu; Tel.: +1-419-530-2167; Fax: +1-419-530-7946

**Keywords:** tautomeric equilibria, IEF-PCM/B97D/aug-cc-pv(q+(d))z, Monte Carlo, solution structure, hydrogen bonds

## Abstract

This follow-up paper completes the author’s investigations to explore the in-solution structural preferences and relative free energies of all OH-substituted oxazole, thiazole, isoxazole, and isothiazole systems. The polarizable continuum dielectric solvent method calculations in the integral-equation formalism (IEF-PCM) were performed at the DFT/B97D/aug-cc-pv(q+(d))z level for the stable neutral tautomers with geometries optimized in dichloromethane and aqueous solution. With the exception of the predictions for the predominant tautomers of the 3OH isoxazole and isothiazole, the results of the IEF-PCM calculations for identifying the most stable tautomer of the given species in the two selected solvents agreed with those from experimental investigations. The calculations predict that the hydroxy proton, with the exception for the 4OH isoxazole and 4OH isothiazole, moves preferentially to the ring nitrogen or to a ring carbon atom in parallel with the development of a C=O group. The remaining, low-fraction OH tautomers will not be observable in the equilibrium compositions. Relative solvation free energies obtained by the free energy perturbation method implemented in Monte Carlo simulations are in moderate accord with the IEF-PCM results, but consideration of the Δ*G*_solv_/MC values in calculating Δ*G*^s^_tot_ maintains the tautomeric preferences. It was revealed from the Monte Carlo solution structure analyses that the S atom is not a hydrogen-bond acceptor in any OH-substituted thiazole or isothiazole, and the OH-substituted isoxazole and oxazole ring oxygens may act as a weak hydrogen-bond acceptor at most. The molecules form 1.0−3.4 solute−water hydrogen bonds in generally unexplored numbers at some specific solute sites. Nonetheless, hydrogen-bond formation is favorable with the NH, C=O and OH groups.

## 1. Introduction

Tautomeric equilibration of suitable molecules is of large practical importance both in chemical syntheses and in general, where intermolecular interactions play a crucial role [1,2]. Drug design is one of the fields where this phenomenon may critically appear [3,4]. The internally minimum-free-energy tautomeric form may not assure the most negative free energy for the drug-receptor system. In order to reach optimal electrostatic and van der Waals interactions and matching of the non-polar sites for the partners [5], the formation of an alternative tautomeric species is needed. Although drug-protein intermolecular hydrogen bonds can be favorably formed in this structure, transformation into this species will increase, in parallel, the internal free energy of the drug component in the complex. The theoretical design process tries to optimize the resultant of these two opposite trends, which needs a thorough exploration of the free energy expense of the tautomerization in condensed phase.

Tautomerization may mean a quite basic restructuring of the drug/ligand molecule, including even ring formation/disruption [3]. In the simplest cases, however, it means only a proton repositioning on the ring atoms or between a ring substituent group and a ring atom. It has been known for a long time that the preferable molecular structure in the isolated phase (practically in the gas phase) may change in a condensed phase or the tautomeric composition is shifted at least in solution. Such shifts have been demonstrated by Moriyasu et al. [6] in a series of experiments considering a number of different solvents when the keto-enol tautomerism of some β-dicarbonyl compounds was followed. On the theoretical side, such studies for aliphatic systems were performed mainly for acetylacetone in aqueous solution [7,8,9]. Proton repositioning is common for five-member aromatics with O and N ring atoms and with OH [10,11,12,13,14,15] or methyl substituents [16]. Theoretical studies for further systems showing proton jumps in intermolecular hydrogen bonds and for species allowing alternative protonation on ring nitrogens were recently reviewed in [2,17].

Structural consequences of the replacement of an oxygen atom by a sulfur atom have been recently studied by the author [18,19]. This issue may become important in drug design since the hydrogen-bond acceptor capacity of the O and S atoms are different. Furthermore, since the X–C and X–N bond lengths are considerably larger with X=S than with X=O, the increased ring size may affect the fit of the ligand to the receptor. In the case where the ligand causes double-binding to the receptor, the position of the second binding site of the ligand changes upon the replacement of O by S, resulting in the increase or decrease of the binding strength.

**Figure 1:**
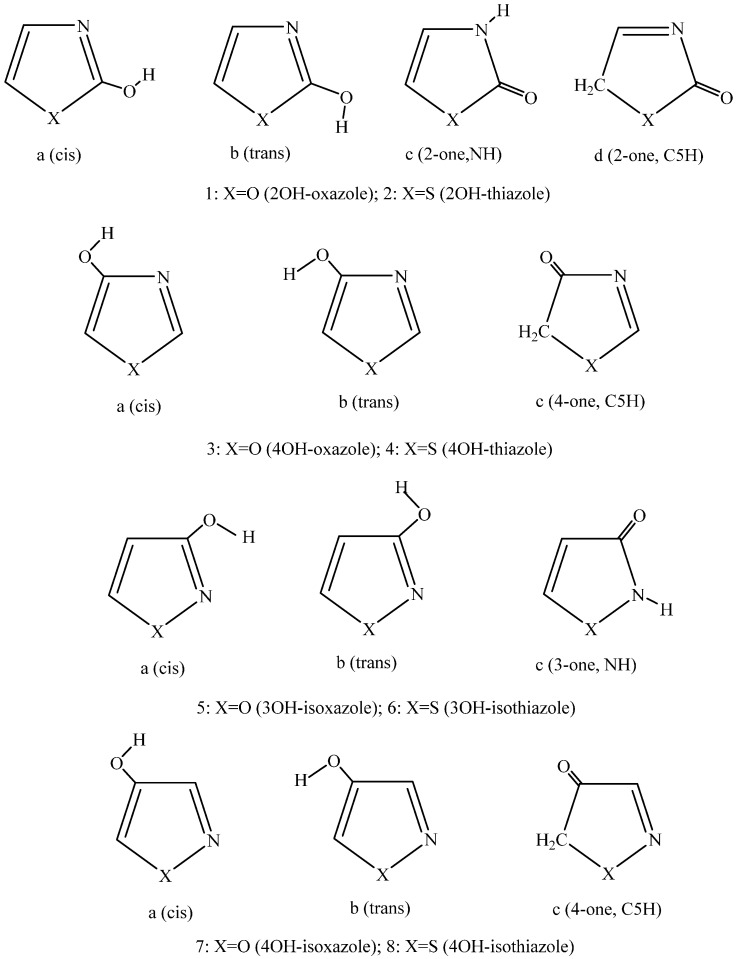
Schematic representation of the structures 1a– 8c in the text.

For OH-substituted thiazoles, Barrett reported a review [20] that the keto-enol tautomerism, thus the repositioning of the OH proton to some ring atoms, largely depends on the presence and quality of other ring substituent(s), which are generally alkyl and/or aromatic groups. This finding was confirmed by Täuscher et al. [21] upon their X-ray studies for differently substituted 4-OH thiazoles. Dahlke and Cramer [22] performed theoretical calculations on the tautomeric composition for the 4a, 4b, and 4c structures (Figure 1) in the gas phase and in aqueous solution by applying multi-coefficient correlation and multi-coefficient Gaussian-3 methods [23,24] and the SM5.R42 semiempirical solvation model. The predominance of the 4a structure with 89% in the gas phase changed upon solvation and the aqueous solution composition moved to 54:23:23 for 4c:4a:4b.

In [19], the present author investigated the performances of the DFT B97D/aug-cc-pv(q+(d))z and the ab initio CCSD(T)/complete basis set levels in combination with the IEF-PCM solvation models for the 5OH derivatives of the rings in Figure 1. Whereas the DFT method predicted fair tautomer compositions in comparison with the experimental result for the 3Me,5OH and 4Me,5OH isoxazole, the ab initio method failed to predict the change of the 5-one, NH and 5-one, C4H preferences for the two methyl derivatives. Furthermore, the ab initio method generally overestimated the free energy difference for the tautomers. Accordingly, the present paper applies only the DFT method for exploring the structures of the different 2, 3, and 4OH derivatives of the title compounds. In the discussion section, results for the 5OH derivative [19] will also be considered.

To our best knowledge, the present investigation is the first high-level theoretical study for these OH-substituted thiazoles and isothiazoles in two solvents and their comparison with the corresponding oxazole and isoxazole species.

## 2. Results and Discussion

### 2.1. Methods and Energy Calculations

Theoretical calculations were performed by using the Gaussian 09 package [25] (Gaussian Inc., Wallingford, CT, USA) running at the Ohio Supercomputer Center (Columbus, OH, USA). The DFT/B97D method of Grimme [26] was applied utilizing the aug-cc-pvtz and aug-cc-pvqz basis sets [27]. In cases of S-containing rings, five d-functions were added to the basis set on the S atom [28]. The solvent effects were considered by using the IEF-PCM solvent model [29]. The dielectric constant was set to 8.93 and 78.39 for dichloromethane (CH_2_Cl_2_) and water, respectively. For cavity creation around the atoms, scaled Bondi radii were accepted [30,31]. Molecular geometries were optimized in both solvents at the IEF-PCM/B97D/aug-cc-pv(t+(d))z level and each structure was identified as a local energy minimum upon frequency analysis. Relative free energies of the conformers/tautomers (Figure 1) were calculated from IEF-PCM/B97D/aug-cc-pv(q+(d))z single-point energies as

Δ*G*^s^_tot_ = (Δ*E*^s^_int_ + Δ*G*^s^_th_) + (Δ*E*_elst_ + Δ*G*_drc_) ≡ Δ*G*^s^_int_ + Δ*G*_solv_ (1)

Δ*E*^s^_int_ and Δ*G*^s^_th_ stand for the relative internal energy and thermal correction, respectively, in solution. Δ*G*^s^_th_ was calculated in the rigid rotor-harmonic oscillator approach [32]. Δ*E*_elst_ accounts for the relative solute−solvent electrostatic interaction energy (in fact free energy) and Δ*G*_drc_ stands for the relative dispersion-repulsion-cavitation free energy.

Explicit-solvent studies were performed through Monte Carlo (MC) simulations. Applying the BOSS 4.8 software (Yale University, New Haven, CT, USA) [33], the NpT (isobaric-isothermal) ensemble was investigated at *T* = 298 K and *p* = 1 atm. Details of such MC simulations were described in the original papers [34,35,36,37]. Explicit-solvent, pre-equilibrated water and dichloromethane boxes were utilized from the BOSS 4.8 library (Yale University, New Haven, CT, USA) and one solute molecule was implemented in the box in every case. Eventually, the water box was comprised of 506 TIP4P water molecules [34], and for the dichloromethane box (264 solvent molecules) the three-point model of Lim [38] was accepted. The simulations applied preferential sampling and periodic boundary conditions. Cutoffs were set to 1200 pm in the water box, and the solvent-solvent cutoff was increased to 1400 pm (nearly the half of the box edge) in the dichloromethane box. Intermolecular interactions were calculated by means of the all-atom OPLS-AA force field [39,40]. The 12-6 LJ parameters were taken from the program’s library. Atomic charges were derived by the CHELPG fit [41] to the IEF-PCM/B97D/aug-cc-pv(q+(d))z molecular electrostatic potential of the optimized solute in the corresponding solvent. Long-range electrostatic interactions were taken into consideration through Ewald summation [42,43].

The relative solvation free energies were obtained by using the free energy perturbation method (FEP) [44,45] along a non-physical path. The OH proton was gradually annihilated and the proton was gradually developed, in parallel, at a new site of the solute. The procedure is thermodynamically legal because *G*_solv_ is a state function, whose variation depends only on the difference of *G*_solv_ for the starting and the end structures, thus is independent of the transformation path. The FEP calculations were controlled by the transformation coupling parameter, λ, which varied from 0 to 1 throughout the procedure. Choosing a λ and a small Δλ value for a step, 7.5 million configurations were generated in the equilibration phase, which was followed by the generation of a subsequent 7.5 million configurations utilized for averaging and providing Δ*G*_solv_(λ,Δλ,average). Applying double-wide sampling, there were 19 steps needed in aqueous solution in order to keep Δ*G*_solv_(λ,Δλ,average) less than about 2 kJ/mol. For the FEP procedure in dichloromethane, about 10 steps were enough. The SD (standard deviation) for the full transformation was a few tenths of a kJ/mol for any tautomeric pair in any solvent. In every step, the interaction potential parameters, pp (λ), varied as a linear function of the *λ* resulting in pp (λ) = pp (λ = 0) + λ (pp (λ = 1) − pp (λ = 0)). Eventually, Δ*G*_solv_ was calculated as Σ_λ_ Δ*G*_solv_(λ,Δλ,average). For more technical details, see former calculations [2,46,47,48].

The IEF-PCM-based results are summarized in Table 1 and Table 2 in comparison with those obtained for the isolated state (gas phase). With the exception of the 3OH isoxazole and 3OH isothiazole species, the preference has not changed upon solvation, but since the calculated relative free energies have been shifted in the condensed phase, the in-solution composition differs from that in the gas phase. *E*_int_ itself is always less negative for any species in solution than its gas-phase optimized counterpart due to smaller or larger geometric changes and charge polarization for the molecule. As consistently found, when Δ*G*_solv_ is negative the related Δ*G*^s^_int_ = *G*^s^_int_(2) − *G*^s^_int_(1) becomes more positive or less negative compared to the corresponding Δ*G*^g^_int_ value. This is the consequence of the polarization of the solute by the solvent, which distorted the gas–phase structure (2) rather than that for (1) in order to develop more negative *G*(2)_solv_, leading to Δ*G*^s^_int_ > Δ*G*^g^_int_ and Δ*G*_solv_ < 0. In contrast, if Δ*G*_solv_ is positive, the relationship Δ*G*^s^_int_ < Δ*G*^g^_int_ held. This latter inequality could be explained so that tautomer (1), which is more stable in the gas phase, undergoes larger destabilization in solution than tautomer (2), so Δ*E*^s^_int_ decreases in comparison with Δ*E*^g^_int_. According to Table 1 and Table 2, this larger destabilization was always entailed with Δ*G*_solv_ > 0, corresponding to solvations when *G*_solv_(1) is more negative than *G*_solv_(2).

Table 3 shows the comparison of the theoretically predicted prevailing tautomers with those from experiments. The agreement is good with one major exception. (A minor difference is there for the 5OH isoxazole. A NH:C4H ratio was calculated theoretically as 43:57 [19], whereas the experimental finding is 55:45). Theory predicted the preference of the 3-one NH tautomer (5c, 6c in Figure 1) for the 3OH isoxazole and 3OH isothiazole, whereas experimentally the 3OH (*cis* or *trans*) form was identified. Since in all other cases the calculated results for the prevailing form agree with the experimentally preferred in-solution species, this single exception is at least surprising. Table 2 shows that the *cis* 3OH tautomer is dominant in the gas phase both with X=O and S for the rings (Figure 1), thus the in-solution predicted result is most likely a consequence of the applied PCM solvation model. The 3-one tautomer is less stable by 4.6 kJ/mol than the *cis* 3OH isoxazole or *cis* 3OH isothiazole in the gas phase. Upon solvation in the IEF-PCM framework, the preference changes to the 3-one, NH form with Δ*G*^s^_tot_ –3.1 to –4.7 kJ/mol for the isoxazole derivative in the two solvents. Even larger free energy shift was calculated for the 3OH isothiazole on the favor of the 3-one, NH form. These results suggest that the applied approximation for the solvation is responsible for the theoretically predicted shift. 

A well-known shortcoming of the PCM method is that it underestimates the effects of the solute−solvent hydrogen bonds on the relative free energies of conformers/tautomers. In Table 4, results of the PCM and MC-based relative solvation-free energies are compared. In the explicit solvent simulations, the effects of the solute−solvent hydrogen bonds are expected to have been considered. FEP sometimes predicts very good relative solvation-free energies [45], whereas they apparently overestimate it in other cases [19]. For the present case, however, the two relative solvation-free energy methods provide fairly close values, as revealed from Δ*G*_solv_ for the 5a → 5c transformation. Both methods favor the solvation of the 3-one, NH form predicting the shift of the tautomeric preference from the *cis* 3OH form to the 3-one, NH structure. This computational result indicates either the failure of both the PCM and MC solvation calculations for the 3OH derivatives or the experiment should be re-interpreted.

For three other MC calculations available both in dichloromethane and water, however, the signs of the Δ*G*_solv_ values are opposite. Nonetheless, consideration of the Δ*G*_solv_/MC values instead of the Δ*G*_solv_/PCM values in equation 1 for the calculations of Δ*G*^s^_tot_ would not reverse the stability order for the corresponding tautomers, although it would slightly affect the calculated compositions. The opposite signs for Δ*G*_solv_ by the two methods for 1c → 1d and 2c → 2d in water would cause changes in the Δ*G*^s^_tot_ of 13–14 kJ/mol if the Δ*G*_solv_/MC were used. Consideration of such a Δ*G*_solv_/MC value, so different from Δ*G*_solv_/PCM, in equation 1 has still no qualitative effect: the stability of the 2-one, NH form relative to the 2-one, C5H species further increases both for oxazole and thiazole.

Table 4 shows that Δ*G*_solv_/PCM is always negative for the transformation toward the tautomer with the larger dipole moment. Thus the signs for Δ*G*_solv_/PCM are apparently governed by the difference in the dipole moments. The conclusion for Δ*G*_solv_/MC must be much more complicated. MC favored the smaller dipole for 1c → 1d, 2c → 2d, in both solvents, and for 8b → 8c in dichloromethane. While the preference of 1c over 1d and that of for 2c over 2d may stem from a weak, hydrogen-bond-like interaction between the N-H bond and the solvent’s Cl atoms, no clear explanation has been found for the preference of the 8c form over 8b in this solvent. The positive Δ*G*_solv_/MC for 1c → 1d and 2c → 2d in water clearly suggests that the solute−solvent hydrogen bonds dominate the calculated Δ*G*_solv_ by MC. It reveals from the 1a → 1c, 2a → 2c, 5a → 5c and 6a → 6c results that the HN–C=O substructure is much more preferably hydrated than the N=C–OH moiety in the case when there are both hydrogen-bond donor and acceptor sites on solute. These results were valid both for the PCM and MC solvations and may be the origin of the calculated 3-one, NH tautomeric preference over the experimentally found 3OH oxazole and thiazole form. Interpretation of the MC solvation becomes more complicated by noting that Δ*G*_solv_/MC is negative for 3b → 3c but is positive for 7b → 7c and 8b → 8c. In these two latter cases the dipole moments conspicuously decrease and the polar site is O–C–C–N in contrast to O–C–N in 3b and 3c.

Overall, the PCM and MC relative solvation free energies correlate weakly at most for OH-substituted oxazole, thiazole, isoxazole and isothiazole. Differences of the Δ*G*_solv_ terms amount to about 8 kJ/mol, but increase to 13–16 kJ/mol for a → c transformations. Nonetheless, the deviations do not cause modifications of the stability order of the tautomers, although the shifts in Δ*G*^s^_tot_ (Equation (1)) result in changes in the theoretical tautomer compositions. Consideration of the MC results is, however, important mainly in aqueous solution to evaluate the underestimated effects of the solute−solvent hydrogen-bond formation, which is an inherent feature of the PCM method.

### 2.2. Equilibration Mechanism

As discussed in detail in [19], the proton relocation between far-lying donor and acceptor sites of the solute is problematic in a non-protic solvent, but a straightforward catalytic mechanism can be devised in solvents with both H-donor and H acceptor sites like in water and methanol. For example, by considering the tautomerization of the 5OH hydroxy species to the O=C and N–H form, the tautomerization may take place in two subsequent steps as follows (The symbol HO–(ringN) stands for the neutral 5OH species, and “ringN” refers for the rest of the solute):

H_2_O + HO–(ringN) ↔ H_3_O^+^ + ^−^O–(ringN) and then ^–^O–(ringN) + H_3_O^+^ ↔ O=C–(ringNH) + H_2_O

or

HO–(ringN) + HOH ↔ HO–(ringNH)^+^ + OH^−^ and then OH^−^ + HO–(ringNH)^+^ ↔ H_2_O + O=C–(ringNH)

The protic solvent takes up the 5-OH proton in the first mechanism, and after several proton jumps along the water network around the solute, this extra proton will be dropped at the H-acceptor site of the solute. When the water takes up the proton, an ion pair of the H_3_O^+^ cation and a solute anion is formed, which is stabilized temporarily by the surrounding water molecules. The neutralization of the system takes place when the extra proton has been forwarded to the H-acceptor site of the solute.

An alternative tautomerization route is when a water protonates the H-acceptor site of the solute and an OH^−^…soluteH^+^ ion pair comes into existence. The direction of the proton current along the water network remains the same as before, and the subsequently formed OH^−^ ions take up the closest water proton and become neutralized. The eventually formed OH^−^ ion facing the solute’s 5–OH group takes the proton of the latter and allows the formation of a C=O group in the N–H tautomer. The question is: which route dominates?

For predicting the mechanism, the HSAB theory (hard-and-soft-acids-and-bases) [53] and its advanced version by introducing a unified picture and the maximum hardness (MH) principles [54,55,56] could be useful. In these papers, attempts were made to assign scales as hard (H) and soft (S) acids and bases. The basic idea was that the formation of the H–H pair is preferred over the H–S combination, thus the favored direction of a reaction in a system of H1–S1 and S2–H2 is the formation of the H1–H2 and S2–S1 pairs. This principle is applicable to the present problem by recognizing that the initial step of the proton repositioning can be considered as an acid−base reaction. Both the water and the solute can be considered as forming an acid + base or a base + acid pair and the starting interaction depends on which assignment corresponds better to the H–H qualification or closer to it.

The scales were calculated on the basis of reaction enthalpies [53] or using maximum hardness parameters derived upon DFT-calculated electronegativity and atomic radii [55]. Use of the MH indices may distinguish the reaction paths above, although their determination for the present molecules would take extremely long calculations. For this reason, only the theoretical possibility has been raised here. Furthermore, the published MH indices in [55] are very similar for some Lewis acids as well as for Lewis bases and it is then becomes questionable whether such small differences would be significant in predicting the favorable tautomerization route for the present proton repositioning.

### 2.3. Solution Structure Simulations

Although it was concluded in the previous section that the FEP/MC calculation of the relative solvation-free energy is not superior compared to the application of the IEF-PCM method, the immediate solvent structure around the solute can be explored only within the framework of an explicit-solvent method. The related results are summarized in Table 5.

Coordination numbers (CN) were calculated by integration of the solute site/solvent site radial distribution functions (rdf) [57] until their first minima. Such rdfs were studied for expected solute−solvent hydrogen bond-forming sites in aqueous solutions. Formations of possibly strong O_ring_/H_w(ater)_ H-bonds were predicted only for isoxazole OH derivatives as concluded from the CN values of 1−2 for 5a and 7b (Table 5), and for the *cis* 5OH and 5-one, C4H tautomers with CN = 1 for each of them [19]. In contrast, no S-H_w(ater)_ hydrogen-bond formation was predicted either for the 2OH and 4OH thiazole, or the 3OH and 4OH isothiazole tautomers. In combination with the findings from the previous study for the 5OH derivatives [19], no remarkable S-H_w(ater)_ hydrogen-bond formation is to be expected for any OH-substituted thiazole and isothiazole ring. This is one of the most important conclusions of the present series studying the effects of the substitution of the ring O atom by an S atom.

The N/H_w_, NH/O_w_, OH/O_w_, and =O/H_w_ coordination numbers are at least of 1 with a few exceptions. Reduced H_w_-binding capacity of the nitrogen atom for 2-one, C5H (1d) can be concluded from the low N/H_w_ coordination number and similarly, although at a lower scale, for the 4-one, C5H (3c) species. This finding is confusing when considering the fairly large dipole moments and the exposure of the nitrogen atoms for H-bond formation. A possible explanation may be in both cases that the neighboring carbonyl oxygen is a better hydrogen-bond acceptor and the water molecule connecting to this oxygen along one of its lone pairs exerts steric hindrance against the development of an N…H_w_ bond. Furthermore, the N atomic charge changed to −0.66 acu (atomic charge unit) in the 1d tautomer from −0.71 acu in the *cis* 2OH oxazole (1a). These atomic charges were fitted to the corresponding in-solution electrostatic potentials of the optimized solute tautomers. The derived charge sets reproduced well the exact DFT dipole moments for any studied species, so they are expected to appropriately reflect the charge polarization in the solute. (In fact, this latter statement is not necessarily true, because atomic charges are not physical entities and the results of the fits depend on the applied fitting method. Theoretically, different charge sets could be derived, which would reproduce the exact dipole moment.).

The large variation of the N/H_w_ values is even more remarkable for the 8b and 8c tautomers of 4OH isothiazole. This difference must reflect large deviations of the derived atomic charges. Indeed, the in-solution electrostatic potential fitted atomic charge for the nitrogen atom is −0.50 and −0.35 acu for the trans 4OH isothiazole (8b) and the 4-one, C5H (8c) tautomer, respectively.

The –O/H_w_ coordination number is typically about 0.7 from MC simulations for any previously studied system, whereas the =O/H_w_ values are generally about 2 or more (see, e.g., [2,46,58]). This experience has been confirmed in the present investigations, as well. The two, fairly low =O/H_w_ values for the 7c and 8c tautomers must be related to their conspicuously low dipole moments in water, which results in a weak solvent-hydrogen localizing ability and generates the smallest numbers of the solute−solvent hydrogen bonds, *n*_HB_.

The *n*_HB_ values (Table 5) scatter in the range of 1.3–3.2 units. The range indicates very meaningful differences in the capacities of the solutes to strongly localize solvent molecules at their hydrogen-bonding sites. The *n*_HB_ values were obtained by integrating the solute−solvent pair-energy distribution functions (pedfs) until their first minima. The minima were sometimes well developed, but could also be poorly visible, or even only an inflection point appeared due to the overlap of the contributions of the more and less strongly localized solvent molecules. When the minimum was well resolved, it appeared generally at *E* = −16.7 kJ/mol (see the *E* value in parentheses in Table 5). For solutes poorly localizing the solvent molecules, the pedf was rather stretched and the minimum moved to −10.5 kJ/mol for 6a. Although the calculated *n*_HB_ was 2.8, this number includes contributions from less strongly localized water molecules due to the most stretched integration range. When the integration limit was found to be the most negative *E* value of −16.7 kJ/mol (1c, 1d, 3c, 2c, 2d, 5c, 7b, 6c, 8b, 8c), *n*_HB_ solvent molecules were considered strongly bound and accordingly formed hydrogen bonds. It is clearly seen from the table that the sums of the CN values are always larger than the calculated corresponding *n*_HB_ values for the given tautomer. This is a general conclusion from the NpT MC calculations utilizing the OPLS-AA force field and the statistics provided by the BOSS 4.8 software [33]. The interpretation of this finding is that thermal disordering moves sometimes the solvent molecules out of their most favorable hydrogen-bonding positions. Although they still remain in the first hydration shell of the solute site and increase the CN value, their individual interaction energies with the solute become less negative than the integration limit value appearing on the pedf as its first minimum.

Since Σ CN > *n*_HB_, one cannot precisely identify the number of the H-bonds at a specific site. In the fortunate case, when the difference is small, a likely assignment can be made. For example, almost all water hydrogens around N and =O are in H–bond to these acceptors in 5c. Similarly, one water oxygen in the NH/O_w_ hydration shell and about 2.6 water hydrogens form altogether 3.5 hydrogen bonds to 3-one, NH isothiazole (6c). Since bifurcated hydrogen bonds are rare in aqueous solution, about 3.5 water molecules can be hypothesized around the O=C–N–H moiety on the basis of the calculated CN and *n*_HB_ values.

The logic leading to the above assignment can be applied, albeit only rarely. The experience from former studies and the inspection of snapshots from simulations suggest that there are about two =O…H_w_ bonds in acids, ketones, and aldehydes. Aliphatic amines form a stable N…H_w_ bond and any OH is a strong H-bond donor [2]. Other hydrogen bonds are less stably maintained.

A large CN may not guarantee the large number of hydrogen bonds to that site. For example, the O_ring_/H_w_ CN is 1.9 for 3OH isoxazole. The footnote in Table 5 indicates that the minimum of the O_ring_/H_w_ rdf was found at 275 pm for 5a. At such a large O_ring_…H_w_ separation no remarkable stabilization should be expected, thus automatic consideration of the CN values could lead to serious misinterpretation of the H-bond pattern of the solute. For the same molecule, a large coordination number was obtained from the integration of the N/H_w_ rdf with the minimum at 270 pm. Within such extended first hydration shells, a number of water hydrogens can reside fairly far from the ring H-bond acceptor sites. They do not form a hydrogen bond but still contribute to CN. Not surprisingly, the *n*_HB_ value is only 2.5 with a stretched pedf (E = −14.6 kJ/mol), whereas Σ CN = 5.6 >> 2.5.

Thus the above analysis suggests that not even the isoxazole ring oxygen is a strong hydrogen-bond acceptor, and oxazole oxygen is barely a hydrogen-bond acceptor. Replacement of the ring O by a sulfur atom would then entirely cancel the H-bond formation at this site of the ring. The weak or extremely weak H-bond-forming capacities of O and S in the rings may be the consequence of the electron-withdrawing effect of the other ring heteroatom and the involvement of an O or S lone pair in the delocalization of the 6π electrons, maintaining the aromatic character of these five-member heterocycles. The calculated, generally 2−3 solute−solvent hydrogen bonds are most likely formed between solvent molecules and the ring nitrogen and the carbonyl oxygen (=O) of the tautomers. The hydrogen-bond analysis finds all OH tautomers as forming about two or more hydrogen bonds with CN = 1 for OH/O_w_, but the OH forms generally do not appear in aqueous solution with the exception of the 4OH isoxazole and 4OH isothiazole species.

## 3. Conclusions

This paper, which is a follow-up study regarding the tautomerism of the 5OH oxazole, thi azole, isoxazole, and isothiazole species [19], completes the author’s investigations for exploring the in-solution structural preferences and relative free energies of all OH-substituted systems with rings indicated above. Calculations utilizing the integral-equation formalism of the polarizable continuum dielectric solvent method (IEF-PCM) were performed at the DFT/B97D/aug-cc-pv(q+(d))z level for the stable neutral tautomers with geometries optimized in dichloromethane and aqueous solution. With the exception of the predictions for the predominant tautomers of the 3OH isoxazole and 3OH isothiazole, the results of the IEF-PCM calculations for identifying the most stable tautomer of the given species in the two selected solvents agreed with those from experimental investigations. The deviation was attributed to the polarizable continuum dielectric solvation model. Applying the free energy perturbation method in explicit-solvent Monte Carlo simulations, the theoretical prediction was maintained. Thus the author speculates whether both the MC and PCM calculations fail for the 3OH species, whereas PCM works correctly for all other system, or the interpretation of the in-solution experiments for assigning the favorable 3OH isoxazole and 3OH isothiazole should be reconsidered.

The IEF-PCM calculations predict for other OH-substituted species that the hydroxy proton generally moves to the ring nitrogen or to a ring carbon atom in parallel with the development of a C=O group. As a result, the remaining low fraction of the OH tautomer will not be observable experimentally in the equilibrium composition. Exception to this proton repositioning was calculated only for the 4OH isoxazole and 4OH isothiazole where the OH tautomer is the major species in equilibrium. FEP/MC relative solvation free energy calculations are in moderate accord with the PCM results, but utilizing the Δ*G*_solv_/MC values in place of Δ*G*_solv_/PCM in calculating Δ*G*^s^_tot,_ the tautomer preferences are maintained.

It was revealed from the Monte Carlo solution-structure analysis that the S atom may be only an extremely weak hydrogen-bond acceptor in any OH-substituted thiazole or isothiazole. The OH-substituted isoxazole ring oxygen may act as a weak hydrogen-bond acceptor, whereas the oxazole derivatives are even less able to localize a water hydrogen in their first hydration shells. All molecules form 1.0−3.4 solute−water hydrogen bonds (including the 5OH derivatives) with generally unexplored number(s) of the H-bonds at some specific sites. The best predictions for the H-bonds may be made as about 1 NH/O_w_ + 2.2 (=O/H_w_) for 3-one, NH isoxazole, about 1 NH/O_w_ + 2.5 (=O/H_w_) for 3-one, NH isothiazole, and about 1 NH/O_w_ + 2.3 (=O/H_w_) for 5-one, NH isothiazole. The OH-containing tautomers should be also good hydrogen-bond donors, but the theoretical calculations predicted observable fractions for them in the equilibrium compositions only for the 4OH isoxazole and 4OH isothiazole species.

## Figures and Tables

**Table 1:** Relative free energies for the conformers/tautomers of the oxazole and thiazole OH derivatives. Energy terms in kJ/mol relative to the corresponding *cis* OH species ^a^.

	Gas	CH_2_Cl_2_			Water		
	Δ*G*^g^_int_	Δ*G*^s^_int_	Δ*G*_solv_	Δ*G*^s^_tot_	Δ*G*^s^_int_	Δ*G*_solv_	Δ*G*^s^_tot_
2OH oxazole							
2OH, *trans*	7.8	10.0	−6.6	3.4	11.2	−9.1	2.1
2-one, NH	−52.0	−45.4	−17.0	−62.4	−42.7	−21.9	−64.6
2-one, C5H	−23.6	−16.8	−20.6	−37.4	−13.4	−26.8	−40.2
2OH thiazole							
2OH, *trans*	12.6	17.1	−11.2	5.9	18.8	−11.5	7.3
2-one, NH	−45.9	−40.7	−14.2	−54.9	−38.2	−14.7	−52.9
2-one, C5H	−9.1	−1.8	−17.8	−19.6	1.6	−19.8	−18.2
4OH oxazole							
4OH, *trans*	11.5	15.1	−10.8	4.3	16.9	−14.4	2.5
4-one, C5H	−29.5	−23.6	−16.3	−39.9	−21.0	−20.8	−41.8
4OH thiazole							
4OH, *trans*	11.9	16.2	−11.6	4.6	18.3	−15.6	2.7
4-one, C5H	−8.0	−1.7	−16.5	−18.2	1.3	−21.8	−20.5

^a^ The free energy components for the oxazole and thiazole OH derivatives were calculated at the B97D/aug-cc-pvqz//B97D/aug-cc-pvtz and B97D/aug-cc-pv(q+d)z//B97D/aug-cc-pv(t+d)z theoretical levels, respectively. The in-solution data were obtained by utilizing the IEF-PCM continuum dielectric solvent approximation. Δ*G*_int_ = Δ*E*_int_ + Δ*G*_thermal_, superscripts “g” and “s” refer to the gas-phase and the in-solution relative terms.

**Table 2:** Relative free energies for the conformers/tautomers of the isoxazole and isothiazole OH derivatives. Energies in kJ/mol relative to the corresponding *cis* OH species ^a^.

	Gas	CH_2_Cl_2_			Water		
	Δ*G*^g^_int_	Δ*G*^s^_int_	Δ*G*_solv_	Δ*G*^s^_tot_	Δ*G*^s^_int_	Δ*G*_solv_	Δ*G*^s^_tot_
3OH isoxazole							
3OH, *trans*	13.5	17.9	−12.7	5.2	20.1	−17.2	2.9
3-one, NH	4.6 ^b^	9.8	−11.2	−3.1 ^b^	11.3	−14.3	−4.7 ^b^
3OH isothiazole							
3OH, *trans*	14.9	19.3	−12.9	6.4	21.6	−17.5	4.1
3-one, NH	4.6	13.6	−19.2	−5.6	16.4	−24.7	−8.2
4OH isoxazole							
4OH, *trans*	0.0	0.1	−0.2	−0.1	0.1	−0.2	−0.1
4-one, C5H	1.9	−0.6	6.6	6.0	−1.6	9.2	7.6
4OH isothiazole							
4OH, *trans*	−0.4	0.8	−1.1	−0.3	1.0	−1.6	−0.6
4-one, C5H	12.9	12.7	3.3	15.9	12.3	4.8	17.1

^a^ See the footnote of Table 1; ^b^ –*RT* ln 2 = –1.71 kJ/mol is included for the entropy of mixing of the optical antipodes with C_1_ symmetry.

**Table 3:** The prevailing tautomers for the OH derivatives in solution with relative free energy of at least 4.184 kJ/mol ^a^.

	CH_2_Cl_2_	Water	Exp ^b,c^
**2OH**			
oxazole	2-one, NH	2-one, NH	2-one, NH
thiazole	2-one, NH	2-one, NH	2-one, NH
**3OH**			
isoxazole	3-one, NH/3OH *cis*	3-one, NH	Mainly 3OH in all media
isothiazole	3-one, NH	3-one, NH	3OH
**4OH**			
oxazole	4-one, C5H	4-one, C5H	4-one, C5H
thiazole	4-one, C5H	4-one, C5H	4OH and 4-one, C5H no ratio provided
isoxazole	4OH, *trans/*4OH, *cis*	4OH, *trans/*4OH, *cis*	4OH
isothiazole	4OH, *trans/*4OH, *cis*	4OH, *trans/*4OH, *cis*	4OH
**5OH** ^d^			
oxazole	5-one, C4H	5-one, C4H	5-one, C4H (in crystal)
thiazole	5-one, C4H	5-one, C4H	
isoxazole	5-one, C4H/5-one, NH	5-one, C4H/5-one, NH	5-one, C4H in CH_3_Cl_3_, NH/CH 55:45 in water ^e^
isothiazole	5-one, NH	5-one, NH	5-one, NH ^f^
3CH_3_,5OH isoxazole		5-one, C4H	C4H/NH 70:30 in water
4CH_3_,5OH isoxazole		5-one, NH	5-one, NH

^a^ If the difference between the most stable and the second-most stable species is less than 4.184 kJ/mol, the code of the second-most stable form appears after the slash; ^b,c^ [49,50]. For the original references see “e” and “f” specifically; Theoretical results from ^d^ [19], ^e^ [51], ^f^ [52].

**Table 4:** Comparison of the relative solvation-free energies from IEF-PCM and Monte Carlo calculations. Corresponding dipole moments are indicated ^a^.

	CH_2_Cl_2_			Water		
	PCM	MC	DM	PCM	MC	DM
Oxazole						
1a → 1c	−17.0		0.25, 6.21	−21.9	−22.4	0.35, 6.52
1c → 1d	**−3.6**	**0.7**	6.21, 7.35	**−4.9**	**9.1**	6.52, 7.71
3b → 3c	−5.5		3.35, 5.15	−6.4	−7.6	3.54, 5.43
Thiazole						
2a → 2c	−14.2		0.58, 5.59	−14.7	−23.7	0.62, 5.90
2c → 2d	**−1.8**	**0.3**	5.59, 7.08	**−5.1**	**8.1**	5.90, 7.48
4b → 4c	−4.9		3.51, 5.33	−6.2	−5.8	3.75, 5.70
Isoxazole						
5a → 5c	−11.2	−9.9	2.31, 4.88	−14.3	−17.7	2.43, 5.14
7b → 7c	6.8		4.46, 0.89	9.4	8.1	4.67, 0.92
Isothiazole						
6a → 6c	−19.2		1.73, 5.34	−24.7	−40.3	1.84, 5.69
8b → 8c	**4.4**	**−1.9**	4.27, 2.05	6.4	5.8	4.51, 2.17

^a^ Energies in kJ/mol, IEF-PCM/B97D/aug-cc-pv(q+(d))z dipole moments along the transformations in Debye. Δ*G*_solv_ with opposite sign from PCM and MC in bold face.

**Table 5:** Coordination numbers (CN) and numbers of solute−water hydrogen bonds ^a^.

	O_ring_/H_w_	S_ring_/H_w_	N/H_w_	NH/O_w_	−O/H_w_	OH/O_w_	=O/H_w_	*n*_HB_(E)
Oxazole								
1a	--- ^b^		1.4		0.6	1.0		2.1(−14.6)
1c	0.3 ^c^			1.1			2.3	2.8(−16.7)
1d	0.1 ^c^		0.6				1.9	2.1(−16.7 ^d^)
3b	0.4		1.3		0.8 ^e^	1.0		2.6(−14.6)
3c	0.1 ^f^		0.9				1.9	2.4(−16.7)
Thiazole								
2a		--- ^b^	1.3 ^g^		0.7	1.0		1.7(−14.6)
2c		--- ^b^		1.0			2.1	2.7(−16.7)
2d		--- ^b^	1.0				1.9	2.2(−16.7 ^d^)
4b		--- ^b^	1.6		0.9	1.0		2.7(−14.6)
4c		--- ^b^	1.0				2.1	2.8(−14.6)
Isoxazole								
5a	1.9 ^h^		2.0 ^i^		0.7	1.0		2.5(−14.6)
5c	--- ^b^			1.0			2.4	3.2(−16.7)
7b	1.1		1.7		0.8	1.0		2.2(−16.7)
7c	0.7 ^j^		1.1				1.5	2.2(−12.6 ^d^)
Isothiazole								
6a		(0.2) ^k^	1.5 ^g^		0.9	1.0		2.8(−10.5)
6c		--- ^b^		1.0			2.6	3.5(−16.7)
8b		--- ^b^	1.5		0.9	1.0		1.9(−16.7 ^d^)
8c		--- ^b^	0.4 ^l^				1.7	1.3(−16.7)

^a^ Coordination numbers were determined by the integration of the corresponding radial distribution functions up to their first minima generally at 250 ± 10 pm. Values in parentheses for *n*_HB_ stand for the integration limits in kJ/mol for the solute−solvent pair-energy distribution functions; ^b^ No local minimum of rdf below 250 pm; ^c^ End of a rdf plateau at 225 pm; ^d^ End of a pedf plateau; ^e^ Rdf minimum at 230 pm; ^f^ Rdf minimum at 220 pm; ^g^ Rdf minimum at 265 pm; ^h^ Rdf minimum at 275 pm; ^i^ Rdf minimum at 270 pm; ^j^ End of a rdf plateau at 245 pm; ^k^ 0.2 H_w_ around the sulfur atom up to S…H_w_ = 250 pm without having a local rdf minimum up to this limit; ^l^ End of a rdf plateau at 240 pm.
